# A little is better than none: the biggest gain of physical activity in patients with ischemic heart disease

**DOI:** 10.1007/s00508-020-01767-x

**Published:** 2020-12-01

**Authors:** Alexandra Huber, Stefan Höfer, Hugo Saner, Neil Oldridge

**Affiliations:** 1 Department of Medical Psychology, Medical University of Innsbruck, Christoph-Probst-Platz 1, 6020 Innsbruck, Austria; 2 Preventive Cardiology and Sports Medicine, Clinic for Cardiology, Inselspital Bern, University of Bern, Bern, Switzerland; 3 College of Health Sciences, University of Wisconsin-Milwaukee, Milwaukee, WI USA

**Keywords:** Health-related quality of life, Physical activity, Ischemic heart disease, Cardiac rehabilitation

## Abstract

**Background:**

There is a relationship between physical activity and health-related quality of life (HRQL) in healthy people and in patients with ischemic heart disease (IHD). The purpose of this study was to determine whether this relationship between sports or recreational physical activity levels and HRQL has a dose-response gradient in patients with IHD.

**Methods:**

Using one generic and three IHD-specific HRQL questionnaires, differences in HRQL scores (adjusted for confounders) were determined for physically a) inactive vs. active patients and b) inactive vs. patients being active 1–2, 3–5, or >5 times per week.

**Results:**

Data were provided by 6143 IHD-patients (angina: *N* = 2033; myocardial infarction: *N* = 2266; ischemic heart failure: *N* = 1844). Regardless of diagnosis or instrument used, when patients were dichotomized as either inactive or active, the latter reported throughout higher physical and emotional HRQL (all *p* < 0.001; *d* = 0.25–0.70). When categorized by physical activity levels, there was a positive HRQL dose-response gradient by increasing levels of physical activity that was most marked between inactive patients and those being active 1–2 times per week (63 82%). Conclusions: Using generic and IHD-specific HRQL questionnaires, there seems to be an overall dose-dependent gradient betweenincreasing levels of sports or recreational physical activity and higher HRQL in patients with angina, myocardial infarction, and ischemic heart failure. The greatest bang for the public health buck still lies on putting all the effort in changing sedentary lifestyle to at least a moderate active one (1–2 times per week), in particular in cardiac rehabilitation settings.

## Introduction

“A little is better than none”. According to Blair et al. [1] this should be the public health message, with evidence of a decreasing health risk gradient with regular physical activity, encouraging the “most sedentary … to become at least moderately active” [2]. At about the same time, Rejeski et al. pointed out that “some positive change in a number of HRQL domain scores does occur when people elect to be more physically active” [3]. More recent data confirm that being physically active is associated with higher health-related quality of life (HRQL) in healthy adults [4–7] with more limited evidence in clinical populations, such as patients with cancer or heart disease [5, 8].

In the last decade, some observational and survey studies [9–15], randomized clinical trials [16–18] and evaluations of cardiac rehabilitation programs [19–21] reported a positive association between HRQL and physical activity in patients with ischemic heart disease (IHD). Nine reports identified the recruited patients as having coronary heart disease [9, 10, 12–14, 16–19], one meta-analysis included IHD patients who also had a myocardial infarction (MI) or angina [20], and the others included patients having a specific diagnosis like MI [11, 15] or ischemic heart failure [21]. These studies used 50-50 generic [10–13, 18, 19] and IHD-specific HRQL questionnaires [9, 14–17, 21], applied different strategies to measure physical activity: instruments with validation [11, 16–19], without validation [9, 10, 13, 15], both [12, 14] or with objective parameters [21], and included mostly cardiac patients in general. Therefore, the robustness of the evidence for a physical activity and HRQL dose-response gradient in patients with IHD is limited.

The HeartQoL project [22] provided an opportunity to respond to these limitations, analyzing data with one generic HRQL questionnaire: the Short-form 36 Health Survey (SF-36 v1) [23], and three IHD-specific HRQL questionnaires: the Seattle angina questionnaire (SAQ) [24], the Minnesota living with heart failure questionnaire (MLHF) [25], and the MacNew questionnaire (MacNew) [26]. Physical activity levels were assessed in terms of frequency with a dichotomous (yes/no) and a 4-level variable (none, 1–2, 3–5, >5 days/week) in 6143 IHD patients (angina: *N* = 2033; myocardial infarction: *N* = 2266; ischemic heart failure: *N* = 1844).

The aim of this study was to examine if there would be a positive dose-response gradient between increasing levels of sports or recreational physical activity and higher HRQL scores on different validated HRQL questionnaires in patients with angina, MI, or ischemic heart failure. In particular, the authors wanted to check the accuracy of the statement “a little is better than none” in terms of physical activity across the three major IHD diagnoses with various instruments in a large international sample.

## Methods

The HeartQoL project was designed to develop an IHD-specific core HRQL questionnaire in patients with IHD who were recruited in 22 countries worldwide [22]. Patients with documented angina (Canadian Cardiovascular Society class II, III, or IV), MI between 1 and 6 months previously, or with ischemic heart failure (New York Heart Association class II, III, or IV, left ventricular dysfunction, and an ejection fraction <40%) were eligible for the HeartQoL project. With site-specific ethics committee approval for the HeartQoL project, all eligible patients provided written informed consent.

### Measures

#### Short-form 36 Health Survey (SF-36, v1)

The SF-36 is a validated 36-item generic health survey summarized as physical component summary (PCS) and mental component summary (MCS) measure with higher norm-based standardized T‑values (mean 50 ± 10) indicating higher HRQL [23]. In this study, scores were estimated using standard (U.S.-derived) scoring algorithms which are recommend for purposes of multinational studies with many countries involved here [27]. Internal consistency was calculated with Cronbach’s alpha for PCS = 0.91 and for MCS = 0.89 in the study population.

#### Seattle angina questionnaire (SAQ)

The SAQ is a validated 19-item, functional status questionnaire designed for patients with IHD [24, 28] with five domain scales: physical limitations, angina stability, angina frequency, treatment satisfaction, and disease perception. Item scores are transformed to scales of 0–100 points with lower scores indicating worse levels of functioning. Only the SAQ physical limitation scores (9 items, score 1–6) were analyzed in terms of Cronbach’s alpha = 0.91 in the study population.

#### Minnesota living with heart failure questionnaire (MLHF)

The MLHF is a validated 21-item HRQL questionnaire designed for patients with heart failure [25], which is summarized as physical (8 items, score 0–40) and emotional dimension (5 items, score 0–25) with a global score (21 items, score 0–105) and lower scores indicating less dysfunction. Cronbach’s alpha was 0.92 (physical dimension), 0.86 (emotional dimension), and 0.93 (global scale) in this study.

#### MacNew questionnaire (MacNew)

The 27-item MacNew questionnaire is validated in patients with angina, MI, and heart failure [29]. The MacNew consists of a physical (13 items), emotional (14 items) and social (13 items) subscale with partly overlapping items which are summarized in a global scale with scores ranging from 1 to 7 (lower scores indicating worse HRQL) [29, 30]. Internal consistency was calculated with Cronbach’s alpha for the physical = 0.91, the emotional = 0.94, and the global scale = 0.95 in this sample.

#### Physical activity

All patients completed two items in the sociodemographic questionnaire which asked whether or not they participated in regular sports or recreational physical activity; if answered positively, they were then asked how often they participated, i.e., 1–2, 3–5, or >5 times/week [22].

### Statistical analysis

Descriptive statistics (means, standard deviations, and frequencies) were used to illustrate the sample sociodemographic data and diagnoses. SF-36, SAQ, MLHF, and MacNew scores were adjusted for sex, age, disease severity, and number of comorbidities as potential confounders. With physical activity as independent and HRQL as dependent variable, analyses of covariance with Bonferroni correction and effect sizes (Cohen’s *d* ≥ 0.20 = small; ≥0.50 = medium; ≥0.80 = large effect) were used to test for HRQL score differences by activity level (active vs. inactive, and inactive vs. 1–2, 3–5, or >5 times/week) with each HRQL questionnaire. All analyses were carried out using SPSS 22 [31].

## Results

### Patient characteristics

#### Sociodemographic and clinical characteristics (Table 1)

**Table 1:** Sociodemographic characteristics in the total group and in patients with angina, myocardial infarction, and ischemic heart failure (data missing if sample sizes do not equal *n* or 100%)

	Total cohort	Angina	Myocardial infarction	Ischemic heart failure
	*N* = 6143 100%	*N* = 2033 33.1%	*N* = 2266 36.9%	*N* = 1844 30.0%
*Age* (years ± *SD*)	62.4 ± 11.3	63.0 ± 10.2	59.6 ± 11.4	65.1 ± 11.5
*Sex* (*N*)				
Female	1520; 24.7%	561; 27.6%	544; 24.0%	415; 22.5%
Male	4620; 75.2%	1471; 72.4%	1722; 76.0%	1427; 77.4%
*Family status *(*N*)				
Single	721; 11.7%	225; 11.1%	272; 12.0%	224; 12.1%
Married	4562; 74.3%	1519; 74.7%	1673; 73.8%	1370; 74.3%
Other	780; 12.7%	248; 12.2%	298; 13.2%	234; 12.7%
*Education* (*N*)				
<High school	2242; 36.5%	763; 37.5%	742; 32.7%	737; 40.0%
High school	1899; 30.9%	649; 31.9%	699; 30.8%	551; 29.9%
>High school	1761; 28.7%	526; 25.9%	747; 33.0%	488; 26.5%
*Risk factors *(*N*)				
Hypertension^a^	3442; 56.0%	1308; 64.3%	1149; 50.7%	985; 53.4%
Diabetes^a^	1290; 21.0%	464; 22.8%	349; 15.4%	477; 25.9%
High cholesterol^a^	3696; 60.2%	1381; 67.9%	1323; 58.4%	992; 53.8%
Current smoker	921; 15.0%	269; 13.2%	377; 16.6%	275; 14.0%
BMI (mean ± SD)	27.4 ± 4.97	27.9 ± 5.02	27.0 ± 4.68	27.3 ± 5.21
*Physical activity *(*N*)				
Not active	2976; 48.7%	972; 48.0%	962; 42.6%	1042; 56.8%
1–2 times/week	1272; 20.8%	431; 21.3%	502; 22.2%	339; 18.5%
3–5 times/week	1294; 21.2%	434; 21.5%	567; 25.1%	293; 16.0%
>5 times/week	573; 9.4%	186; 9.2%	228; 10.1%	159; 8.7%

*BMI* body mass index, *MI* myocardial infarction, *N* number of patients, *SD* standard deviation

^a^As told by physician

The cohort in this HeartQoL project substudy consisted of 6143 patients with IHD (angina: *N* = 2033; MI: *N* = 2266; ischemic heart failure: *N* = 1844). Sociodemographic and cardiac risk factor details are provided in Table 1. Being physically inactive was reported by 48.7% of all patients, while the others were at least active 1–2 times per week. Patients with ischemic heart failure were most likely to be inactive (56.8%) (Table 1).

### Physical activity levels and health-related quality of life

#### Generic health-related quality of life: SF 36 (Table 2)

**Table 2:** Short-form 36 (SF-36) health-related quality of life; mean ± standard deviation in the total cohort and in patients (*N*) with angina, myocardial infarction, or ischemic heart failure by level of physical activity (inactive vs. active); scores adjusted for gender, age, disease severity, and number of comorbidities

	SF-36; M ± SD (*N*)		
	PCS	MCS	*p*-value/Cohen’s *d*
**Total cohort (*N*** **=** **4845)**			
Inactive	35.7 ± 9.8 (2329)	45.4 ± 11.1 (2329)	PCS: *p* < 0.001/*d* = 0.70 MCS: *p* < 0.001/*d* = 0.28
Active	42.6 ± 9.8 (2516)	48.4 ± 10.6 (2516)	
*Activity levels*			
Inactive^a^	35.7 ± 9.8 (2329)	45.4 ± 11.1 (2329)	PCS: all *p* < 0.001/*d*: a vs. b = 0.55; a vs. c = 0.63; a vs. d = 0.65 MCS: *p* < 0.001/*d*: a vs. b = 0.22; a vs. c = 0.31; a vs. d = 0.41; *p* = 0.042/*d:* b vs. d = 0.19
1–2 times/week^b^	41.0 ± 9.6 (1012)	47.7 ± 10.7 (1012)	
3–5 times/week^c^	41.9 ± 9.8 (1029)	48.6 ± 10.5 (1029)	
>5 times/week^d^	42.2 ± 10.1 (461)	49.7 ± 10.3 (461)	
**Angina (*N*** **=** **1723)**			
Inactive	35.3 ± 9.6 (805)	45.2 ± 10.8 (805)	PCS: *p* < 0.001/*d* = 0.49 MCS: *p* < 0.001/*d* = 0.25
Active	40.0 ± 9.5 (918)	47.9 ± 11.0 (918)	
*Activity levels*			
Inactive^a^	35.3 ± 9.6 (805)	45.2 ± 10.8 (805)	PCS: all *p* < 0.001/*d*: a vs. b = 0.48; a vs. c = 0.50; a vs. d = 0.46 MCS: *p* < 0.001/*d*: a vs. c = 0.28; *p* = 0.003/*d:* a vs. d = 0.36
1–2 times/week^b^	39.9 ± 9.5 (365)	47.0 ± 11.0 (365)	
3–5 times/week^c^	40.1 ± 9.5 (392)	48.2 ± 11.0 (392)	
>5 times/week^d^	39.7 ± 9.6 (158)	49.1 ± 10.8 (158)	
**Myocardial infarction (*N*** **=** **1543)**			
Inactive	40.0 ± 9.3 (650)	45.5 ± 11.2 (650)	PCS: *p* < 0.001/*d* *=* 0.61 MCS: *p* < 0.001/*d* = 0.27
Active	45.5 ± 8.7 (893)	48.4 ± 10.3 (893)	
*Activity levels*			
Inactive^a^	40.0 ± 9.3 (650)	45.5 ± 11.2 (650)	PCS: all *p* < 0.001/*d*: a vs. b = 0.52; a vs. c = 0.64; a vs. d = 0.75 MCS: *p* < 0.001/*d*: a vs. c = 0.28; a vs. d = 0.40; *p* = 0.002/*d*: a vs. b = 0.21
1–2 times/week^b^	44.6 ± 8.5 (353)	47.8 ± 10.3 (353)	
3–5 times/week^c^	45.8 ± 8.7 (376)	48.5 ± 10.4 (376)	
>5 times/week^d^	46.9 ± 9.1 (162)	49.7 ± 9.9 (162)	
**Ischemic heart failure (*N*** **=** **1579)**			
Inactive	32.9 ± 9.2 (874)	45.4 ± 11.4 (874)	PCS: *p* < 0.001/*d* = 0.61 MCS: *p* < 0.001/*d* = 0.34
Active	38.7 ± 9.8 (705)	49.1 ± 10.4 (705)	
*Activity levels*			
Inactive^a^	32.9 ± 9.2 (874)	45.4 ± 11.4 (874)	PCS: all *p* < 0.001/*d*: a vs. b = 0.54; a vs. c = 0.62; a vs. d = 0.69 MCS: all *p* < 0.001/*d*: a vs. b = 0.28; a vs. c = 0.36; a vs. d = 0.45
1–2 times/week^b^	38.0 ± 9.7 (294)	48.5 ± 10.8 (294)	
3–5 times/week^c^	38.8 ± 9.9 (261)	49.3 ± 10.0 (261)	
>5 times/week^d^	39.5 ± 9.9 (141)	50.3 ± 10.4 (141)	

*p*-value: analyses of covariance with Bonferroni correction

*N* number of patients; *M* mean; *SD* standard deviation; *d* Cohen’s *d*

^a^ inactive; ^b^ 1–2 times/week; ^c^ 3–5 times/week; ^d^ >5 times/week

The SF-36 was completed by 4845 patients (angina: *N* = 1723; MI: *N* = 1543; ischemic heart failure *N* = 1579).

##### Physically active versus inactive

Physically active patients in the total group and in each diagnosis reported higher PCS and MCS scores (higher HRQL) than inactive patients (all *p* < 0.001). The associated PCS effect size in the total cohort was *d* = 0.70 (angina *d* = 0.49, MI *d* = 0.61, ischemic heart failure *d* = 0.61), whereas the total MCS effect size was lower *d* = 0.28 (angina *d* = 0.25, MI *d* = 0.27, ischemic heart failure *d* = 0.34).

##### Levels of physical activity (times/week)

Higher PCS and MCS scores were associated with increasing levels of physical activity in the total group and in each diagnosis. In the total cohort, patients who were physically active 1–2, 3–5, and >5 times/week had higher PCS (*p* < 0.001; *d* = 0.55–0.65) and MCS (*p* < 0.001; *d* = 0.22–0.41) scores than inactive patients. In particular, patients with MI or ischemic heart failure had a higher physical and emotional benefit from more physical activity units. Associated PCS and MCS effect sizes for each diagnosis are shown in Table 2 with PCS effect sizes being consistently larger (*d* = 0.46–0.75) than MCS effect sizes (*d* = 0.21–0.45).

#### Specific health-related quality of life: SAQ, MLHF, and MacNew (Tables 3 and 4)

**Table 3:** Seattle angina questionnaire (SAQ) and Minnesota living with heart failure (MLHF) health-related quality of life; mean ± standard deviation in the total cohort and in patients (*N*) with either angina or ischemic heart failure by level of physical activity (inactive vs. active); scores adjusted for gender, age, disease severity, and number of comorbidities

	SAQ; M ± SD (*N*)	MLHF; M ± SD (*N*)		
	Physical limitation	Global	Physical	Emotional
**Angina (*N*** **=** **1831)**				
Inactive	57.7 ± 21.6 (873)	–	–	–
Active	68.5 ± 20.9 (958)			
*p*-value/Cohen’s *d*	*p* < 0.001/*d* = 0.51			
*Activity levels*				
Inactive^a^	57.7 ± 21.6 (873)	–	–	–
1–2 times/week^b^	66.9 ± 20.8 (383)			
3–5 times/week^c^	70.4 ± 20.7 (403)			
>5 times/week^d^	67.6 ± 21.2 (166)			
*p*-value/Cohen’s *d*	All *p* < 0.001/*d*: a vs. b = 0.43; a vs. c = 0.60; a vs. d = 0.46			
**Ischemic heart failure (*N*** **=** **1685)**				
Inactive	–	40.7 ± 21.5 (947)	19.1 ± 10.0 (947)	7.6 ± 6.3 (944)
Active		29.5 ± 20.5 (738)	13.1 ± 9.8 (738)	5.6 ± 5.5 (736)
*p*-value/Cohen’s *d*		*p* < 0.001/*d* *=* 0.53	*p* < 0.001/*d* *=* 0.61	*p* < 0.001/*d* = 0.34
*Activity levels*				
Inactive^a^	–	40.7 ± 21.5 (947)	19.1 ± 10.0 (947)	7.6 ± 6.3 (944)
1–2 times/week^b^		32.0 ± 21.8 (314)	14.2 ± 10.0 (314)	6.1 ± 5.8 (313)
3–5 times/week^c^		28.5 ± 19.5 (269)	12.7 ± 9.5 (269)	5.4 ± 5.3 (268)
>5 times/week^d^		25.6 ± 19.0 (144)	11.4 ± 9.2 (144)	4.8 ± 4.9 (144)
*p*-value/Cohen’s *d*		All *p* < 0.001/*d*: a vs. b = 0.40; a vs. c = 0.60; a vs. d = 0.75	All *p* < 0.001/*d*: a vs. b = 0.49; a vs. c = 0.66; a vs. d = 0.80	*p* < 0.001/*d*: a vs. c = 0.38; a vs. d = 0.50; *p* = 0.004/*d*: a vs. b = 0.25

*p*-value: analyses of covariance with Bonferroni correction

**Table 4:** MacNew health-related quality of life; mean ± standard deviation in the total cohort and in patients (*N*) with angina, myocardial infarction, or ischemic heart failure by level of physical activity (inactive vs. active and inactive); scores adjusted for gender, age, disease severity, and number of comorbidities

	MacNew; M ± SD (*N*)		
	Global	Physical	Emotional
**Total cohort (*N*** **=** **5039)**			
Inactive	4.7 ± 1.1 (2442)	4.5 ± 1.2 (2442)	4.9 ± 1.1 (2440)
Active	5.3 ± 1.0 (2597)	5.2 ± 1.1 (2594)	5.3 ± 1.1 (2596)
*p*-value/Cohen’s *d*	*p* < 0.001/*d* = 0.57	*p* < 0.001/*d* = 0.61	*p* < 0.001/*d* = 0.36
*Activity levels*			
Inactive^a^	4.7 ± 1.1 (2442)	4.5 ± 1.2 (2442)	4.9 ± 1.1 (2440)
1–2 times/week^b^	5.2 ± 1.0 (1050)	5.2 ± 1.1 (1048)	5.2 ± 1.1 (1048)
3–5 times/week^c^	5.3 ± 1.0 (1054)	5.3 ± 1.1 (1054)	5.4 ± 1.0 (1055)
>5 times/week^d^	5.4 ± 1.0 (472)	5.4 ± 1.1 (471)	5.5 ± 1.0 (472)
*p*-value/Cohen’s *d*	*p* < 0.001/*d:* a vs. b = 0.48; a vs. c = 0.57; a vs. d = 0.67; *p* = 0.020/*d*: b vs. d = 0.20; *p* = 0.047/*d*: b vs. c = 0.10	*p* < 0.001/*d*: a vs. b = 0.61; a vs. c = 0.70; a vs. d = 0.78; *p* = 0.039/*d*: b vs. c = 0.09	*p* < 0.001/*d*: a vs. b = 0.27; a vs. c = 0.48; a vs. d = 0.57; *p* = 0.005/*d*: b vs. d = 0.29; *p* = 0.050/*d*: b vs. c = 0.19
**Angina (*N*** **=** **1758)**			
Inactive	4.7 ± 1.0 (828)	4.5 ± 1.2 (828)	4.8 ± 1.1 (828)
Active	5.2 ± 1.0 (936)	5.1 ± 1.1 (934)	5.2 ± 1.1 (935)
*p*-value/Cohen’s *d*	*p* < 0.001/*d* = 0.50	*p* < 0.001; *d* = 0.52	*p* < 0.001; *d* = 0.36
*Activity levels*			
Inactive^a^	4.7 ± 1.0 (828)	4.5 ± 1.2 (828)	4.8 ± 1.1 (828)
1–2 times/week^b^	5.1 ± 1.0 (377)	5.0 ± 1.1 (375)	5.2 ± 1.1 (376)
3–5 times/week^c^	5.2 ± 1.0 (394)	5.1 ± 1.1 (394)	5.2 ± 1.1 (394)
>5 times/week^d^	5.2 ± 1.0 (159)	5.1 ± 1.1 (159)	5.2 ± 1.0 (159)
*p*-value/Cohen’s *d*	All *p* < 0.001/*d*: a vs. b = 0.40; a vs. c = 0.50; a vs. d = 0.50	All *p* < 0.001/*d*: a vs. b = 0.43; a vs. c = 0.52; a vs. d = 0.52	All *p* < 0.001/*d*: a vs. b = 0.36; a vs. c = 0.36; a vs. d = 0.38
**Myocardial infarction (*N*** **=** **1621)**			
Inactive	5.0 ± 1.1 (693)	4.9 ± 1.2 (693)	5.0 ± 1.1 (692)
Active	5.5 ± 0.9 (928)	5.5 ± 1.0 (927)	5.5 ± 1.0 (929)
*p*-value/Cohen’s *d*	*p* < 0.001/*d* = 0.50	*p* < 0.001/*d* = 0.55	*p* < 0.001/*d* = 0.48
*Activity levels*			
Inactive^a^	5.0 ± 1.1 (693)	4.9 ± 1.2 (693)	5.0 ± 1.1 (692)
1–2 times/week^b^	5.4 ± 0.9 (361)	5.5 ± 1.0 (361)	5.4 ± 1.0 (361)
3–5 times/week^c^	5.5 ± 1.0 (391)	5.5 ± 1.0 (391)	5.5 ± 1.0 (392)
>5 times/week^d^	5.6 ± 0.9 (172)	5.6 ± 1.0 (171)	5.6 ± 1.0 (172)
*p*-value/Cohen’s *d*	All *p* < 0.001/*d*: a vs. b = 0.40; a vs. c = 0.48; a vs. d = 0.60	All *p* < 0.001/*d:* a vs. b = 0.55; a vs. c = 0.55; a vs. d = 0.64	All *p* < 0.001/*d*: a vs. b = 0.38; a vs. c = 0.48; a vs. d = 0.57
**Ischemic heart failure (*N*** **=** **1654)**			
Inactive	4.6 ± 1.1 (921)	4.3 ± 1.2 (921)	4.8 ± 1.2 (920)
Active	5.1 ± 1.0 (733)	4.9 ± 1.2 (733)	5.2 ± 1.1 (732)
*p*-value/Cohen’s *d*	*p* < 0.001/*d* = 0.48	*p* < 0.001/*d* = 0.50	*p* < 0.001/*d* = 0.35
*Activity levels*			
Inactive^a^	4.6 ± 1.1 (921)	4.3 ± 1.2 (921)	4.8 ± 1.2 (920)
1–2 times/week^b^	5.0 ± 1.0 (312)	4.8 ± 1.2 (312)	5.1 ± 1.1 (311)
3–5 times/week^c^	5.2 ± 1.0 (269)	5.0 ± 1.1 (269)	5.3 ± 1.1 (269)
>5 times/week^d^	5.3 ± 1.0 (141)	5.1 ± 1.1 (141)	5.5 ± 1.0 (141)
*p*-value/Cohen’s *d*	All *p* < 0.001/*d*: a vs. b = 0.38; a vs. c = 0.57; a vs. d = 0.67	All *p* < 0.001/*d*: a vs. b = 0.42; a vs. c = 0.61; a vs. d = 0.70	All *p* < 0.001/*d*: a vs. b = 0.26; a vs. c = 0.45; a vs. d = 0.64

*p*-value: analyses of covariance with Bonferroni correction

*N* number of patients; *M* mean; *SD* standard deviation; *d* Cohen’s *d*

The Seattle angina questionnaire was only completed by patients with angina (*N* = 1831; Table 3).

##### Physically active versus inactive

Higher physical limitation SAQ scores (higher HRQL) were reported by physically active patients than inactive patients (*p* < 0.001; *d* = 0.51).

##### Levels of physical activity (times/week)

Significantly higher SAQ physical limitation scores were reported by patients who were physically active 1–2, 3–5, and >5 times/week when compared to inactive patients (all differences: *p* < 0.001; *d* = 0.43–0.60; Table 3).

The Minnesota living with heart failure questionnaire was only answered by patients with ischemic heart failure (*N* = 1685; Table 3).

##### Physically active versus inactive

Lower global (*d* = 0.53), physical (*d* = 0.61), and emotional (*d* = 0.34) MLHF scores (higher HRQL) were reported by physically active than by inactive patients (all *p* < 0.001).

##### Levels of physical activity (times/week)

Significantly lower global, physical, and emotional MLHF scores were reported by patients being physically active 1–2, 3–5, and >5 times/week when compared to inactive patients (*p* < 0.001–0.004). Associated MLHF global effect sizes for inactive versus increasingly physically active patients with ischemic heart failure ranged from *d* = 0.40 to 0.75 (physical dimension: *d* = 0.49–0.80; emotional dimension: *d* = 0.25–0.50; Table 3).

The MacNew questionnaire was completed by 5039 patients (angina: *N* = 1921; MI: *N* = 2235; ischemic heart failure: *N* = 1781; Table 4).

##### Physically active versus inactive

Physically active patients in the total group and in patients with angina, MI, or ischemic heart failure reported higher global, physical, and emotional MacNew scores (higher HRQL) than inactive patients (all *p* < 0.001). Associated global effect sizes in total were *d* = 0.57 (angina *d* = 0.50, MI *d* = 0.50, ischemic heart failure *d* = 0.48); the corresponding physical subscale effect sizes ranged from *d* = 0.50 to 0.61, and the emotional subscale effect sizes from *d* = 0.35 to 0.48.

##### Levels of physical activity (times/week)

Significantly higher MacNew global, physical, and emotional scores in the total group and in each diagnosis were associated with increasing levels of physical activity when compared to inactive patients (all *p* < 0.001). The associated global (*d* = 0.38–0.67) and subscale effect sizes (physical *d* = 0.42–0.78; emotional *d* = 0.26–0.64) for inactive versus increasingly physically active patients are detailed in Table 4. In the total cohort, small effects were found in patients being physically active >5 times/week, reporting higher global (*d* = 0.20) and emotional subscale (*d* = 0.29) HRQL scores than patients being physically active 1–2 times/week. All other HRQL scores and effect sizes are shown in Table 4.

#### “A little is better than none”? (Fig. 1)

**Fig. 1:**
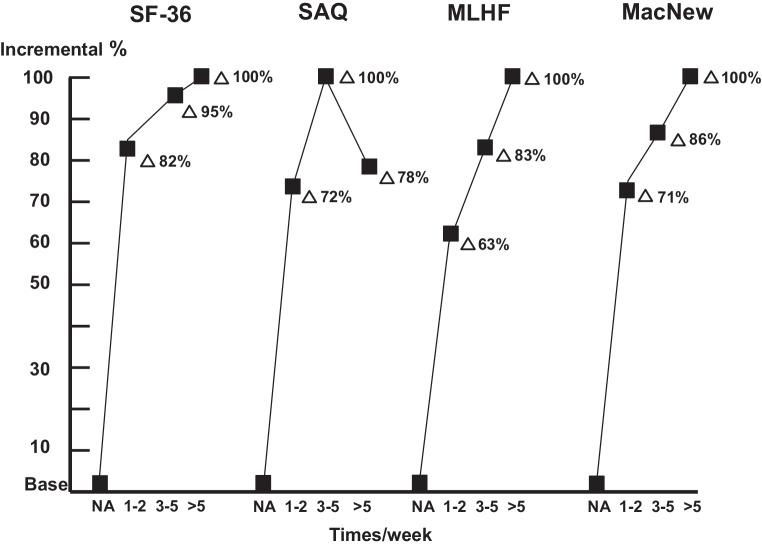
Physical health-related quality of life score differences (scores adjusted for gender, age, disease severity, and number of comorbidities) as a percentage of the largest difference (∆ %) between inactive patients (base, *NA*) and the highest HRQL in active patients (times/week) for the Short Form-36 (*SF-36*), Seattle angina questionnaire (*SAQ*), MacNew, and Minnesota living with heart failure questionnaire (*MLHF*)

As the largest incremental differences (63–82%) in physical HRQL between inactive patients and those who were active either 1–2, 3–5, or >5 times/week, were observed in patients who were active 1–2 times/week (Fig. 1), the question “a little is better than none” can be positively answered based on the empirical evidence found.

## Discussion

Whether sports and recreational physical activity were measured either as a dichotomous or as a four-level variable, physically active patients with angina, MI, or ischemic heart failure consistently reported significantly higher generic and IHD-specific HRQL scores (adjusted for sex, age, disease severity, and the number of comorbidities) than were reported by physically inactive patients in this study. The only exceptions contrary to increasing levels of activity and simultaneously higher HRQL were the SF-36 MCS and SAQ physical limitation scores in patients with angina who were physically active 3–5 times/week. They reported higher physical HRQL and less limitations than patients being active >5 times/week. This might probably be the result of patients with angina limiting activity levels to a more moderate frequency due to chest discomfort when being active more often. Although the cross-sectional nature of the HeartQoL project precludes determination of a causal physical activity and HRQL dose-response relationship, the results in this study provide substantiation of a positive HRQL dose-response gradient in patients with IHD with increasing levels of physical activity levels associated with higher HRQL scores.

A major factor differentiating the findings in the current study examining the relationship between physical activity and HRQL is that data on patients with each of the three major IHD diagnoses, i.e. angina, MI, and ischemic heart failure, are available from the same sample. In contrast, the majority of the studies published in 2010 or later included patients with IHD in general without specifying an exact diagnosis [9, 10, 12–14, 16–19]. Furthermore, the second major difference between this study and the others published in the last decade is that all patients here completed one generic (SF-36) and three IHD-specific core questionnaires (SAQ, MLHF, MacNew) underlining the robustness of the positive HRQL dose-response gradient across generic and specific questionnaires. Finally, all patients provided data on their regular sports or recreational physical activity as both a dichotomous and a multilevel variable.

Regardless of whether physical activity was reported as a dichotomous or multilevel variable, and whether HRQL was assessed with a generic or an IHD-specific questionnaire, all of the HRQL scores and effect sizes between inactive patients and patients being active 1–2, 3–5, and >5 times/week (except for the SF-36 PCS and SAQ in patients with angina) were incrementally higher than or equal to those at the lower physical activity level. As the physical HRQL dimension was common to each of the used questionnaires, these scores were scrutinized for evidence of a gradient between increasing physical activity levels and higher HRQL. The largest incremental differences in physical HRQL between inactive and active patients at different levels were found at an average of 72% in patients who were active at least 1–2 times/week. These observations provide further evidence of a positive dose-gradient between increasing physical activity and higher HRQL in the cardiac cohort as a whole and in each IHD diagnosis reinforcing the earlier observations that doing some activity gives patients “the greatest bang for their HRQL buck”, i.e., essentially that some physical activity is better than none [1–3]. Importantly from a clinical counseling perspective [32], the dose-response gradient between HRQL and increasing levels of physical activity is particularly marked in patients who are active at least 1–2 times/week when compared to inactive patients. This would support physicians and other healthcare professionals when counseling patients to change their lifestyle behavior. Based on the guidelines of the Secondary Prevention and Rehabilitation Section of the European Association of Preventive Cardiology [33], exercise training should follow an individual approach after careful clinical evaluation in patients across a wide spectrum of cardiovascular diseases. General exercise recommendations focus more on the improvement of cardiac patients’ physical capacity, i.e. frequency, intensity, duration, and type of exercise and not on their HRQL. The specific exercise recommendations include a frequency of at least 3 days per week, a moderate intensity of 45–59% of peak oxygen consumption, 50–70% of peak watts, 55–69% of peak heart rate, 4–6 metabolic equivalents or the speech rule, a duration of at least 20–30 min per session, and aerobic, resistance/strength, flexibility, balance, and/or muscle training.

Both the robustness and the frequency of documenting a dose-response relationship between HRQL and physical activity are negatively influenced by the predominance of studies with a cross-sectional design and by the range of instruments used to measure both HRQL and physical activity in general [4] and clinical populations [8]. In this study, limitations include the cross-sectional study design and the limited two-item self-report physical activity levels as well as the 8‑year recruitment time span of patients living in 22 different countries with 15 different languages. Moreover, the presented results are based on less female than male patients (about one quarter of the study sample) implying caution with interpretations. Therefore, future studies should use in-depth tools, such as activity tracking devices, electronic diaries, and experience sampling methods when examining cardiac patients’ physical activity.

On the other hand, regardless of the specific IHD diagnosis and the HRQL questionnaire used, inactive patients reported poorer HRQL, active patients reported higher HRQL, and higher HRQL scores usually reflected an increase in physical activity levels. From a measurement point of view, these data substantially increase the robustness of a positive gradient with increasing physical activity levels and higher HRQL in patients with IHD.

## Conclusion

The relationship between higher HRQL and regular recreational physical activity or sports was shown with both dichotomous (yes/no) and increasing levels of regular physical activity (none vs. 1–2, 3–5, and >5 times/week), with generic and IHD diagnosis-specific HRQL questionnaires, and with each specific IHD diagnosis, i.e. angina, MI, or ischemic heart failure. The greatest bang for the public health buck still lies on putting all the effort in changing sedentary lifestyle to at least a moderate active one such as that recommended in cardiac rehabilitation settings.
